# *Syntrichia laevipila* Brid., a Bryophyta from Northwest Argentina as a Source of Antioxidants and Antimicrobials

**DOI:** 10.3390/plants14020253

**Published:** 2025-01-17

**Authors:** Luis Ignacio Jiménez, Florencia Maria Correa Uriburu, José Javier Martínez Chamás, Guillermo Martin Suárez, Iris Catiana Zampini, Mario J. Simirgiotis, María Inés Isla

**Affiliations:** 1 Instituto de Bioprospección y Fisiología Vegetal (INBIOFIV-CONICET-UNT), San Miguel de Tucumán T4000CBG, Argentina; lijimenez@lillo.org.ar (L.I.J.); florcorreau@gmail.com (F.M.C.U.); jmartinezchamas@gmail.com (J.J.M.C.); zampini@csnat.unt.edu.ar (I.C.Z.); 2 Facultad de Ciencias Naturales e IML, Universidad Nacional de Tucumán (UNT), San Miguel de Tucumán T4000JFE, Argentina; 3 Instituto Criptogámico, Laboratorio de Briología, Fundación Miguel Lillo 251, San Miguel de Tucumán T4000JFD, Argentina; 4 Unidad Ejecutora Lillo (CONICET—Fundación Miguel Lillo), Miguel Lillo 251, San Miguel de Tucumán T4000JFD, Argentina; 5 Instituto de Farmacia, Facultad de Ciencias, Campus Isla Teja, Universidad Austral de Chile, Valdivia 5090000, Chile; mario.simirgiotis@uach.cl

**Keywords:** bryophytes, antioxidant capacity, antimicrobial activity, phytochemical screening, moss-derived bioactives

## Abstract

In recent years, numerous studies have emerged on the biological activities of bryophytes and their potential for therapeutic use. However, mosses appear to be a relatively overlooked group. The objective of this study was to conduct a phytochemical analysis of one hydroalcoholic extract of *Syntrichia laevipila* and to evaluate its potential as an antioxidant and antimicrobial agent. The moss was collected in the Chaco Serrano region of Argentina, specifically on *Jacaranda mimosifolia*, and subsequently extracted by maceration in ethanol/water. UHPLC/ESI/MS/MS analysis identified 32 peaks, including phenolic compounds (phenolic acids, lignans, chalcones, and flavonoids) and non-hydrophilic compounds (terpenoids, fatty acids, and brassinosteroids). Maslinic and oleanolic acids, two triterpenoids present in S. *laevipila,* were also detected in *J. mimosifolia*, a substrate of this moss. The concentration of phenolic compounds was 19.05 ± 0.21 µg GAE/mL, while the total flavonoid concentration was 13.13 ± 0.33 µg QE/mL. The determination of reducing and total sugars yielded 0.22 ± 0.03 mg GE/mL and 1.26 ± 0.24 mg GE/mL, respectively, while the concentration of soluble proteins was 90.60 ± 4.50 µg BSAE/mL. The extract exhibited antioxidant properties by scavenging ABTS^•+^, H_2_O_2_, AAPH, and HO^•^ radicals. Additionally, it demonstrated antibacterial activity by inhibiting the growth of four strains of *Staphylococcus aureus*. The data obtained suggest that the hydroalcoholic extract of *S. laevipila* possesses significant potential as a natural antioxidant and antimicrobial agent, making it a promising candidate for the development of phytotherapeutic and cosmetic products.

## 1. Introduction

Bryophytes are the closest modern relatives to the ancestors of the first plants that succeeded in adapting to life on land approximately 470 to 515 million years ago [1]. They have diversified early into three distinct extant phyla: Marchantiophyta (liverworts), Bryophyta (mosses), and Anthocerotophyta (hornworts). Bryophytes are important components of terrestrial ecosystems that can be found in all climatic regions, both in cool and humid habitats [2]. This is probably a consequence of the poikilohydric nature of bryophytes, meaning that they have a poor capacity to regulate internal water content and thus are passively dependent on ambient water availability [3].

**Figure 1:**
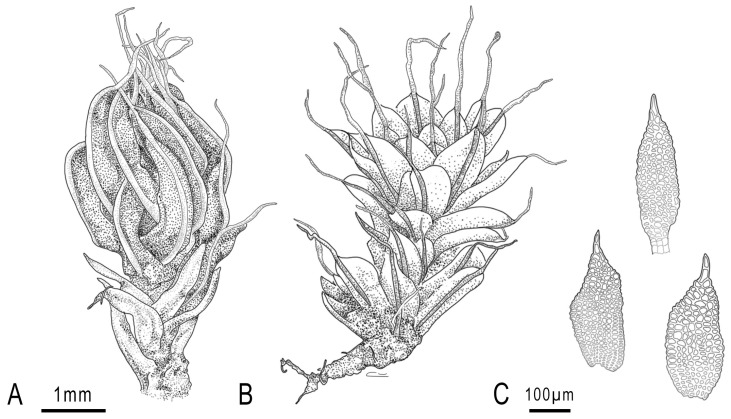
*S. laevipila*, (**A**)—Habit of dry plant, (**B**)—Habit of wet plant, (**C**)—Specialized asexual propagule. The drawing was made by the authors.

More than 2200 secondary or specialized and primary metabolites have been described from bryophytes. The natural products isolated are mainly terpenoids (including mono-, sesqui-, and diterpenoids), flavonoids, (bis)bibenzyls, polysaccharides, rare amino acids, and lipids and derivates [3,4]. The biologically active compounds that can be obtained from bryophytes include phytotoxic, antibacterial, antifungal, antispasmodic, insect antifeedant, and molluscicide compounds; antioxidants; antipyretic, anti-inflammatory, anticancer, and anti-HIV-1 compounds; and neurotrophic compounds [2,4]. *Syntrichia laevipila* Brid. (Pottiaceae) (Figure 1) has been reported from Africa, America, Asia, Australia, Europe, and New Zealand, growing on a wide variety of trees and occasionally on rocks and walls [5]. In the Chaco Serrano Forest from Argentina, it has been found on two native trees, *Ceiba speciosa* (A. St.-Hil.) Ravenna (local name: Palo borracho) and *Jacaranda mimosifolia* D. Don (local name: Jacaranda). The samples grow either purely or in combination with other mosses, such as *Dimerodontium balansae* Müll. Hal. ex Besch., *Tricherpodium beccarii* (Müll. Hal.) Pursell or *Venturiella glaziovii* (Hampe) Pursell. To the best of our knowledge, no specific phytochemical or biological activity studies have been conducted so far. Therefore, the aim of the present study was to determine the phytochemical compounds profile and content of the hydroalcoholic extract obtained from *S. laevipila* collected in the Chaco Serrano Forest from Argentina and evaluate its antioxidant and antibacterial activities on human pathogenic bacteria of clinical interest.

## 2. Results and Discussion

**Figure 2:**
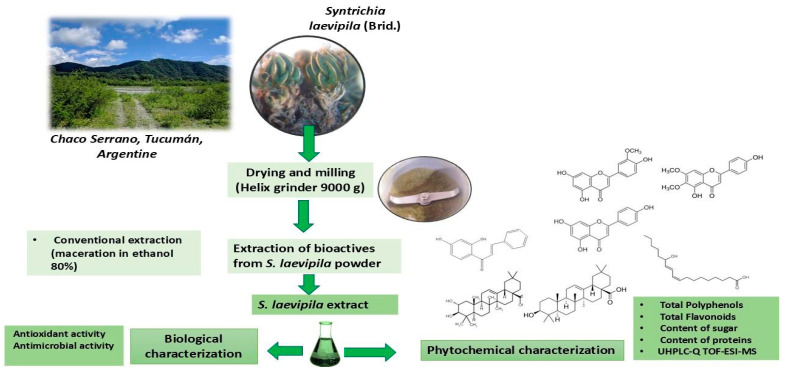
Flowchart of process of obtention of *S. laevipila* extracts and its characterization.

*S. laevipila* collected in Chaco Serrano, Tucumán, Argentina, was used in this study. The dry plant material was ground to obtain a fine powder, and an ethanolic extract was prepared by maceration and chemically characterized and standardized, and its functional properties were determined (Figure 2).

### 2.1. Phytochemical Composition of S. laevipila Extracts

#### 2.1.1. Quantitative Analysis

The phytochemical composition of *S. laevipila* ethanolic extract was analyzed. A higher concentration of total flavonoids was measured in the *S. laevipila* extract (13.13 ± 0.33 µg QE/mL) compared with those previously reported for five bryophytes species, i.e., *Brachythecium rutabulum* (Hedw.) Schimp., *Callicladium haldaneanum* (Grev.) H.A. Crum, *Hypnum cupressiforme* Hedw., *Orthodicranum montanum* (Hedw.) Loeske and *Polytrichastrum formosum* (as *Polytrichum*) (Hedw.) G.L. Sm. (1.31 ± 0.02, 1.03 ± 0.04, 0.68 ± 0.11, 1.12 ± 0.07, 2.12 ± 0.04 µgQE/mL, respectively) [6]. The content of total flavonoids and the other phenolic compounds varies according to their ability to tolerate both biotic and abiotic stress. Some factors, such as UV radiation, temperature, and water deficit, have an important effect on the synthesis of flavonoids [7,8]. In addition, the highest flavonoid content in *S. laevipila* is reasonable, because the genus *Syntrichia* contains some of the most desiccation-tolerant species, and in turn this desiccation tolerance is usually related to mechanisms of tolerance to UV radiation [9]. It has been widely documented that mosses have a high production of flavonoids because they play a significant role in this group of plants [10,11].

Regarding the sugars content, reducing sugars content of *S. laevipila* (0.22 ± 0.03 mg GE/mL) is about six times lower than the total sugar content (1.26 ± 0.24 mg GE/mL). Sucrose was not detected in *S. laevipila,* but the presence of melibiose, a reducing disaccharide formed by an α-1,6 bond between galactose and glucose, was demonstrated by UHPLC/ESI/MS/MS (Table 1). This sugar was also found in the chemical profile of another species of moss, such as *P. formosum* [12]. Several authors have reported that a common characteristic in tissues tolerant to desiccation is a low level of reducing sugars, glucose and fructose [13,14,15,16], and that the reducing sugars content does not vary during dehydration events [17]. It has been suggested that the importance of this low level of reducing sugars is to minimize protein damage resulting from the Amadori and Maillard reactions [18]. In these reactions, glucose and fructose non-enzymatically attack the amino groups of proteins to form glycosylated or fructosylated derivatives. These products can undergo complex interactions with each other to form brown polymeric products (Maillard reaction) [17]. Furthermore, it was reported that the content of reducing sugars decreases during desiccation events, while sugars such as disaccharides remain unchanged. The content of soluble proteins was also low (90.60 ± 4.50 µg BSAE/mL). Bu et al. [19] reported that the content of soluble proteins in mosses of soil biocrusts decreased in response to dehydration and thermal stress events.

#### 2.1.2. Metabolomics in *S. laevipila* Extracts

**Figure 3:**
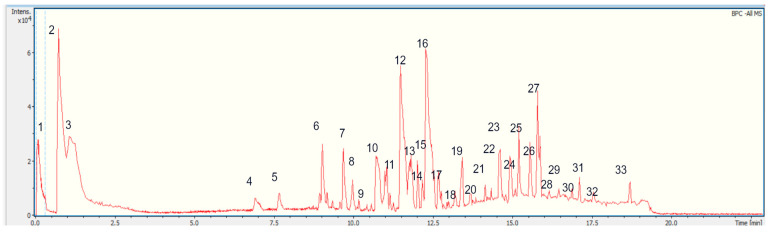
UHPLC/ESI/MS/MS chromatogram of *S. laevipila* extract. The numbers above the peaks correspond to major components identified in the extract.

**Figure 4:**
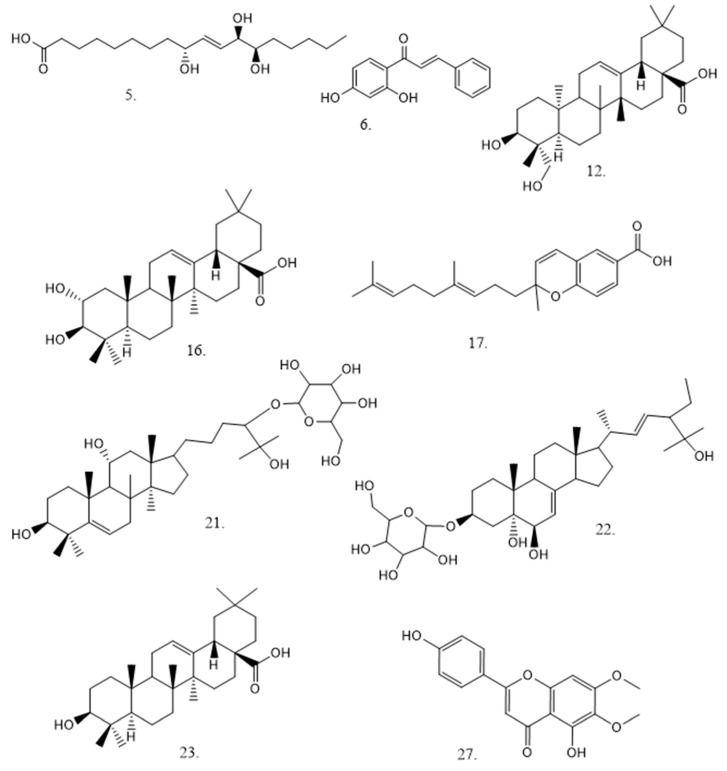
Structures of some representative compounds detected in *S. laevipila*: peak 5, pinellic acid; peak 6, 2’,4’-Dihydroxychalcone; peak 12, hederagenin; peak 16, maslinic acid; peak 17, piperochromenoic acid; peak 21, mogroside I-A1; peak 22, recurvoside A; peak 23, oleanolic acid; and peak 27, cirsimaritin.

Thirty-two peaks (Figure 3; Table 1) were tentatively identified for the first time in *S. laevipila* ethanolic extract using UHPLC/ESI/MS/MS in negative mode. The metabolites identified in this species were mainly phenolic compounds, triterpenoid derivatives, and fatty acids (Table 1; Figure 4). *S. laevipila* was collected from *Jacaranda mimosifolia,* and some of the components previously reported in this plant source are coincident with those reported here. For instance, some compounds identified in the *S. laevipila* extract are also present in the bark of *J. mimosifolia*, such as triterpenoids and phenylpropanoids derived from apigenin [20,21,22,23]. The chemical composition of bryophyte extract could be influenced by territorial factors, as substrate composition on which the moss grows, as well as climatic variations, and this determines their pharmacological potential [24].

**Phenolic compounds**: Several phenolic compounds were found in 300 moss species representing 59 families [25,26]. Phenolic acids, lignans, chalcones, and flavonoids have been found in *S. laevipila* ethanolic extract.

**Phenolic acids**: Peak 7 with daughter ion characteristic of caffeoyl-D-glucose (CG, parent pseudomolecular ion at *m/z*: 341.1041, C_15_H_18_O_9_), was identified. This compound was previously identified in several berries [27] and in the moss *Cryphaea heteromalla* (Hedw.) Brid. [28]. Although biological data on CG is currently lacking due to challenges in isolating and characterizing the compounds, it is anticipated that CG may exhibit numerous health benefits. This is based on the potential of intestinal bacterial esterase and glycosidases to hydrolyze the ester bond, producing caffeic acid, which has been shown to have various functional properties, including antioxidant, anti-inflammatory, immunomodulatory, antimicrobial, neuroprotective, antianxiolytic, antiproliferative, antiobesity, angiotensin-converting enzyme inhibition, and antiglycation activities [29,30,31,32].

**Lignans:** Peak 20 was identified as kadangustin C (ion at *m/z*: 621.23826, C_34_H_37_O_11_). This compound was isolated from several fruit seeds and peels, and bioactivities related to anti-HIV, immunodeficiency, cytotoxicity, and antiproliferative effects were previously reported [33].

**Chalcones:** Two chalcones were identified, 2’,4’-dihydroxychalcone and 2’,3’-dihydroxychalcone, peaks 6 and 8, (parent ions around *m*/*z*: 239.07021, C_15_H_11_O_3_), respectively. 2’,4’-dihydroxychalcone isolated from *Zuccagnia punctata* Cav. exhibits a diverse range of pharmacological effects, including anticancer, antioxidant, antibiotic, antifungal, hypocholesterolemic, and hypoglycemic activities [34].

**Flavonoids:** Several flavonoids were identified, some of them co-spiking with authentic standards: cirsimaritin, apigenin, and chrysoeriol, peaks 27, 29, and 31, (C_17_H_13_O_6_, C_15_H_10_O_5_, and C_16_H_12_O_6_), respectively. These compounds are known to have a variety of therapeutic properties, including antioxidant, anticancer, antiviral, anti-inflammatory, antimutagenic, and antibacterial effects [35,36,37].

**Non-hydrophilic compounds**: non-hydrophilic compounds, including free fatty acids and terpenoids, were identified.

**Terpenoids:** Nine terpenoids were identified in this work. Peaks 11, 12, 13, 15, 16, 21, 22, 23 and 24, were assigned as kallolide B, hederagenin 20(S); asiatic acid, gypsogenin; maslinic acid, mogroside I-A-1; recurvoside A; oleanolic acid and bryonioside A, (formulas: C_13_H_27_O_8_, C_30_H_47_O_4_, C_30_H_47_O_5_, C_30_H_45_O_4_, C_30_H_47_O_4_, C_36_H_31_O_9_, C_35_H_59_O_9_, C_30_H_47_O_3_, C_36_H_59_O_9_) respectively. These compounds exhibit multiple pharmaceutical and biological activities, including antitumor, anti-inflammatory, antidepressant, neuroprotective, hepatoprotective, gastroprotective, hypolipidemic, anti-atherosclerotic, antidiabetic, antileishmanial, antiviral antibacterial, and antifungal activity [38,39,40,41,42]. Maslinic and oleanolic acids were reported from *J. mimosifolia.* bark [20,21,22,23]. Extracts of various parts of *J. mimosifolia* are traditionally used in many countries to cure ulcers and amoebic infections, syphilis, and as an astringent in diarrhea and dysentery. In addition to its broad spectrum of biological features, such as antioxidant, antiulcer, antileishmanial, and antiprotozoal activities [24].

**Fatty acids (FAs):** FAs from bryophytes, including saturated, mono-, polyunsaturated, and acetylenic fatty acids. FAs are usually present as part of membrane phospho- and glycolipids or as constituents in triacylglycerides (TAGs). In *S. laevipila* extract, 9(S),12(S),13(S)-trihydroxy-10*R*-octadecenoic acid (pinellic acid, peak 5), 9,10-dihydroxy-12-octadecenoic acid (peak 10), 13-hydroxy-9Z,11E-octadecadienoic acid (coriolic acid, peak 14), *R*-2-hydroxystearic acid (peak 25), and palmitic acid (peak 26, all ions with their respective formulas: C_18_H_33_O_5_, C_18_H_33_O_4_, C_18_H_31_O_3_, C_16_H_31_O_2_, C_16_H_31_O_2_) were found. The antioxidant, antimicrobial, anti-inflammatory, antiallergic, and cytotoxicity properties were previously demonstrated to compound 1 in other plant material [43,44,45,46]. The compounds derived from the metabolism of linoleic acid were identified for the first time in *S. laevipila*. The compound coriolic acid was isolated previously from *Salicornia herbacea* (L.) L [47]. This compound decreased the transcriptional and translational levels of the c-Myc gene, which is a breast cancer stem cell survival.

**Brassinosteroid (BRs):** 6-deoxocastastasterone and holocastasterone (peaks 28 and 32, C_28_H_47_O_4_, C_29_H_49_O_5_) were identified. In a previous report it was demonstrated that non-flowering land plants can synthesize BRs, including *Marchantia polymorpha* L. (liverwort), *Selaginella moellendorffii Hieron*. (lycophyte), and *Physcomitrella patens* (Hedw.) Bruch & Schimp. (moss) [48]. Although the physiological roles of BRs in lower plants have not yet been established, the result implies that BRs are probably involved in regulating some events in the growth and differentiation of lower plants [49]. This represents, to our knowledge, the first report of BR presence in *S. laevipila*.

### 2.2. Biological Properties

#### 2.2.1. Antioxidant Activity

During the oxidative stress process, reactive species centered in oxygen atoms (ROS), such as hydroxyl radicals and non-radical species such as hydrogen peroxide, are produced. These species can react with a wide range of molecules found in living cells, such as sugars, amino acids, lipids, nucleic acids, and proteins, producing their oxidation and consequently pathological processes or alterations in food or cosmetic products [50].

Several methods are used to measure the antioxidant capacity of natural products and permit them to evaluate their potential use as antioxidants. The ABTS^•+^ assay is a popular, sensitive, and reproducible technique used to evaluate the antiradical potency of extracts by donating hydrogen atoms to form a non-radical molecule [50]. The phenolic compounds concentration values of *S. laevipila* extract required to achieve 50% of radical scavenging capacity, SC_50_, were determined. This magnitude was obtained from the slope of the linear variation in the percentage of radical scavenging (%RS) vs. the phenolic compounds concentration of *S. laevipila* extract. This extract showed high scavenging activity of ABTS^•+^ (SC_50_ 4.38 ± 0.54 μg GAE/mL). Moss extract’s ability to scavenge the ABTS^•+^ was previously reported in other species such as *Philonotis hastata* (Duby) Wijk & Margad [51]. The hydroalcoholic extract of *S. laevipila* also showed scavenging activity of hydroxyl radical with an SC_50_ value of 12.35 ± 0.57 μg GAE/mL. This capacity was previously reported to other moss extracts [51,52]. According to our results, the reactivity of the *S. laevipila* extract to scavenge ABTS^•+^ was higher than to scavenge HO^•^.

Hydrogen peroxide is basically a weak oxidizing agent. It can cross cell membranes and react with ions such as Fe^2+^ to form a hydroxyl radical, which is a strong and toxic oxidizing agent for the cell, so it is necessary to look for compounds that neutralize these oxidizing agents. The analyzed extract was effective in the scavenging of hydrogen peroxide (H_2_O_2_ SC_50_: 5.32 ± 0.51 μg GAE/mL). Although there is growing interest in the antioxidant potential of mosses, research on their ability to neutralize reactive non-radical species is still scarce. At present, only the moss *Thuidium tamariscellum* (Müll. Hal.) Bosch & Sande Lac. is reported to have the ability to scavenge hydrogen peroxide [53].

The erythrocyte membrane contains a large amount of polyunsaturated fatty acids, which makes it vulnerable to oxidative stress processes such as lipid peroxidation. This is a process that plays a key role in oxidative stress in biological systems because it produces membrane alteration and cell damage [53]. Studies have revealed that several plant-derived drugs contain principles that possess the ability to facilitate the stability of biological membranes when exposed to induced lyses. *S. laevipila* extract achieved 50% inhibition of cell lysis produced by AAPH at a concentration of 0.68 ± 0.02 μg GAE/mL. This antioxidant capacity can be compared with natural and synthetic antioxidants used commercially (butylhydroxytoluene (BHT) SC_50_ = 1.20 ± 0.10 µg/mL; quercetin: SC_50_ = 0.90 ± 0.08 µg/mL). Oyedapo et al. [51] found that phenolic-enriched extracts from the moss *P. hastata* inhibited the cellular lysis of red blood cells by 50% (19.19 ± 2.66), evidencing the ability of phenolic compounds, principally flavonoids, to stabilize the erythrocyte membrane.

Pearson correlation (Table 2) showed an association between the scavenging effect of ABTS^•+^, hydrogen peroxide, and hydroxyl radicals with the concentration of total phenolic compounds and flavonoids. Additionally, there is a positive correlation among the different antioxidant tests. Furthermore, the concentration of total phenolic compounds and flavonoids also exhibits a positive association.

In previous reports, antioxidant activity was demonstrated in some phenolic compounds identified in the *S. laevipila* extract, such as 2’,4’-dihydroxychalcone, cirsimarin, apigenin, and terpenoids such as oleanolic acid and maslinic acid [29,31,54,55,56,57,58,59]. For this, these compounds could be responsible for the antioxidant capacity that was found in *S. laevipila* extract.

For this activity, the *S. laevipila* extract could be included in cosmetics, medicinal, or food preparation to protect the preparation from oxidation or for skin care or the body against the effect of free radicals.

#### 2.2.2. Antimicrobial Activity

*Staphylococcus aureus* is a major human bacterial pathogen. This bacterium is widely distributed in the environment and in the normal flora of the skin and mucous membranes of healthy individuals. *S. aureus* does not cause infection on healthy skin; however, if it enters internal tissues, it can produce severe infections. Treatment remains a significant challenge due to the emergence of resistance to multiple antibiotics, such as methicillin resistance [60]. Therefore, the discovery of antibiotic molecules or extracts for use against methicillin-resistant *S. aureus* is very important.

There are no previous reports of the antimicrobial activity of the ethanolic extract of *S. laevipila* against *S. aureus*. However, some authors have reported antibacterial activity only against *Paenibacillus larvae*, by agar diffusion [61].

An initial evaluation of the antimicrobial activity of *S. laevipila* extract was performed using the bioautographic method on two clinical strains of *S. aureus,* one methicillin-resistant and the other methicillin-sensitive, isolated from skin and soft tissue infections, and two ATCC strains. The extract showed notable activity against all tested strains, prompting further investigation to establish the minimum inhibitory concentration (MIC) required to arrest its growth and the concentration needed to achieve a 99.5% reduction in microbial count.

The *S. laevipila* extract showed antimicrobial activity (Table 3), inhibiting the growth of methicillin-sensitive *S. aureus* ATCC 29213 and methicillin-resistant *S. aureus* ATCC 43300 with MIC values of 7.5 µg GAE/mL. The extract proved to be more active against the clinical isolates (*S. aureus* INBIOFIV S1 and INBIOFIV S9) that were antibiotic multiresistant (Table 3), inhibiting its growth at the lowest concentration tested for both strains (MIC: 3.7 µg/mL). Crude extracts that present MIC values below 100 μg/mL [62], are generally accepted as a criterion for selecting antimicrobials with promising properties. The MIC values of the *S. laevipila* extract were 17 times lower than the maximum suggested to be considered potentially antimicrobial.

Currently, there are studies on other species of the genus *Syntrichia*, such as *S. ruralis* (Hedw.) F. Weber & D. Mohr, that demonstrated activity against Gram-negative bacteria [63]. Other authors reported the absence of activity in *S. ruralis* against Gram-negative bacteria and Gram-positive bacteria [64]. The variability in the potency of antimicrobial activity observed in different studies may be attributed to the chemical composition of extracts that are determined by factors such as the collection region and extraction methods, the type of solvent, and the material vegetal/solvent ratio used. It is known that the chemical responses of bryophytes are likely the result of both their evolutionary history and adaptations to their local environment [65]. This variation can even occur among specimens of the same species, depending on their geographical locations and collection dates [66].

Several authors demonstrated a strong antibacterial activity of 2’,4’-dihydroxychalcone, a compound identified in *S. laevipila* extract, against an *S. aureus* strain (strain ATCC 25923) [67,68,69]. Furthermore, hederagenin, cirsimaritin and apigenin were ascribed as antimicrobial on *S. aureus* with different potency in several higher plants [36,70,71].

The results suggest that the presence of 2’,4’-dihydroxychalcone, hederagenin, and cirsimaritin would contribute to the antibacterial properties of the *S. laevipila* extract.

The *S. laevipila* extract provides significant opportunities for newer antibiotic drug discoveries for human health care.

## 3. Materials and Methods

### 3.1. Chemicals, Reagents, and Materials

Folin–Ciocalteau reagent, Bradford reagent, AlCl_3_, FeCl_3_, gallic acid, quercetin, albumin serum bovine, phenol, 4-aminoantipyrene, peroxidase, 2,2′-Azobis(2-methylpropionamidine) dihydrochloride (AAPH), 2-deoxy-D-ribose, EDTA, H_2_O_2_, ascorbic acid, 2-thiobarbituric acid, trichloro-acetic, 3-[4, 5-dimethylthiazol-2-yl]-2, 5-diphenyltetrazolium bromide (MTT) and methanol ≥99.9% were acquired at Sigma Aldrich, St. Louis, MO, USA; silica gel 60 F-254 (0.2 mm) was purchased in Merck, Darmstadt, Alemania. Mueller–Hinton broth (CAMHB) and antimicrobial agents were supplied by Laboratorios Britania S.A., Ciudad Autónoma de Buenos Aires, Argentina.

### 3.2. Plant Material

*S. laevipila* (Figure 1) was collected in Chaco Serrano, Tucumán, Argentina (26°14′53″ S; 65°30′39″ W; 1.126 m ASL, and 26°15′22″ S; 65°32′22″ W; 1.175 m ASL, and 26°14′02″ S; 65°30′25″ W; 1.118 m ASL) on *J. mimosifolia.* The entire plant was collected. The mosses were studied morphologically with the conventional techniques proposed by Zander et al. [72]. The voucher specimen was deposited in the collection INBIOFIV (INBIO 101). The plant material was carefully washed with running water to remove soil particles and adhered plants. The plant material was dried at 40 °C in a forced-air oven until constant weight and then was ground in a Helix mill (Numak, F100 Power 1/2 HP-0.75 Kw, Brusque, Brazil) to obtain a fine powder.

### 3.3. Plant Extract Preparation

Grounded and dried *S. laevipila* (1 g) was extracted in 20 mL of 80% ethanol for 30 min at 40 °C in an ultrasonic bath (Ultrasonic bath Arcano Model PS-10A, Ultrasonic Technology Co., Ltd., Jinan, China). Then, the extract was vacuum filtered and stored at −20 °C until use. A fraction of extract was dried by rotary evaporator (BÜCHI R-110) to use in UHPLC-Q TOF-ESI-MS analysis.

### 3.4. Determination of Chemical Composition

#### 3.4.1. Total Polyphenols and Flavonoids Quantification

The extractive solution was standardized by the determination of total phenolic compound (TPC) content by using Folin–Ciocalteu reagent [73] and total flavonoids (TF) by using the method of Woisky and Salatino [74]. Absorbance was recorded in a UV/visible spectrophotometer (Jasco v-630, Thermo Fisher Scientific, Tokyo, Japan). The calibration curves were performed using gallic acid and quercetin as reference compounds (Supplementary Materials). The phenolic compounds content and flavonoid content were expressed as μg of gallic acid equivalent (GAE) per mL (μg GAE/mL) and quercetin equivalents (QE) per mL (μg QE/mL), respectively.

#### 3.4.2. Reducing and Total Sugars Quantification

Reducing sugars and total sugar were determined using the Somogyi-Nelson method [75,76] and phenol-sulfuric method [77], respectively, for *S. laevipila* powder extractive solution. Absorbance was recorded with a UV/visible spectrophotometer (Jasco v-630, Thermo Fisher Scientific, Tokyo, Japan). The calibration curves were performed using glucose as the reference compound. The results were expressed as glucose equivalents per mL (mg GE/mL).

#### 3.4.3. Soluble Protein Quantification

Soluble proteins were determined by Bradford [78]. Absorbance at 595 nm was recorded with a UV/visible spectrophotometer (Jasco v-630, Thermo Fisher Scientific, Tokyo, Japan). The calibration curves were performed using bovine serum albumin (BSA) as a reference compound. The results were expressed as BSA equivalent per mL (μg BSAE/mL).

#### 3.4.4. UHPLC-Q TOF-ESI-MS

##### LC Parameters and MS Parameters

The separation and identification of the compounds present in the *S. laevipila* extracts were performed on a UHPLC-ESI-QTOF-MS system equipped with UHPLC Ultimate 3000 RS with Chromeleon 6.8 software (Dionex GmbH, Idstein, Germany) and Bruker maXis ESI-QTOF-MS with the software Data Analysis 4.0 (all Bruker Daltonik GmbH, Bremen, Germany). A total of 5 mg of dry extract was dissolved in 2 mL of methanol ≥99.9% and filtered with a polytetrafluoroethylene (PTFE) filter, and 10 µL was injected into the equipment. The chromatographic equipment consisted of a quaternary pump, an autosampler, a thermostated column compartment, and a photodiode array detector. Elution was performed with a binary gradient system with eluent (A) 0.1% formic acid in the water, eluent (B) 0.1% formic acid in the acetonitrile and the gradient: 12% B isocratic (0–1 min), 12–99% B (1–15 min), 99% B isocratic (15–18 min), 99–12% B (18–18.20 min), 12% B (18.20–20 min). Separation was carried out with a Thermo 5 µm C18 80 Å column (150 mm × 4.6 mm) at a flow rate of 0.3 mL/min. ESI-QTOF-MS experiments were recorded in negative ion mode, and the scan range was between 100 and 1200 *m*/*z*. Electrospray ionization (ESI) conditions included a capillary temperature of 200 °C, a capillary voltage of 2.0 kV, a dry gas flow rate of 8 L/min, and a nebulizer pressure of 2 bar. The experiments were performed in automatic MS/MS mode. The structural characterization of specialized metabolites was based on HR full MS, fragmentation patterns, and comparisons with the literature data.

### 3.5. Biological Properties

#### 3.5.1. Antioxidant Activity

##### Total Antioxidant Capacity Assay

The total antioxidant activity of extracts was measured by the improved ABTS radical cation (ABTS^•+^) method as described by Correa Uriburu et al. [79] ABTS^•+^ was mixed with different amounts of extract (1–8 μg GAE). Then, 80% ethanol was used as a negative control. Absorbance was recorded at 734 nm after 6 min with an in-microplate reader (Microplate Reader Thermo Scientific Multiskan GO, Vantaa, Finland). Results are expressed as scavenging concentration of 50% (SC_50_) of ABTS^•+^ expressed as μg GAE/mL.

##### Hydrogen Peroxide (H_2_O_2_) Scavenging

The H_2_O_2_ scavenging was assessed by Fernando and Soysa [80]. Briefly, different concentrations of the extract (3–8 µg GAE/mL) were mixed with 80 μL H_2_O_2_ (0.7 mM), allowing it to stand for 3 min at room temperature. Then, 87.5 μL of phenol (12 mM), 25 μL of 4-aminoantipyrene (0.5 mM), and 15 μL of peroxidase (1.0 U/mL) dissolved in sodium phosphate buffer (84 mM, pH 7.0) were added. It was incubated for 30 min at 37 °C, and the product was determined by recording the absorbance at 504 nm in a microplate reader (Microplate Reader Thermo Scientific Multiskan GO, Vantaa, Finland). Results are expressed as SC_50_ values (μg GAE/mL).

##### Stabilization of Human Red Blood Cell Membrane

The assay was performed according to Orqueda et al. [81]. The protective effect of different concentrations of the *S. laevipila* extract (0.20 to 5 µg GAE/mL) on the red blood cell membrane (5% human red blood cell suspension) by oxidation with 2,2′-Azobis(2-methylpropionamidine) dihydrochloride (AAPH) (200 mM) was tested spectrophotometrically (Jasco v-630, Thermo Fisher Scientific, Tokyo, Japan) at 545 nm under hypotonic conditions. Results are expressed as the inhibitory concentration of 50% of stabilization of red blood cell membranes (IC_50_ values) in μg GAE/mL.

##### Hydroxyl Radical Scavenging Assay

The experiment was performed based on the deoxyribose degradation assay developed by Chobot [82]. The reaction mixture contained *S. laevipila* extract (0.5–10 µg GAE/mL) in KH_2_PO_4_/KOH buffer (pH 7.4), 50 mL of 10.4 mM 2-deoxy-D-ribose, 50 mL of 50 mM FeCl_3_, and 50 mL of 52 mM EDTA. To start the Fenton reaction, 50 mL of 10 mM H_2_O_2_ and 50 mL of 1.0 mM ascorbic acid were added. The reaction mixture was incubated for 1 h at 37 °C. Then, 500 mL of 2-thiobarbituric acid (1%, *w*/*v*) dissolved in 3% (*w*/*v*) trichloroacetic acid was added. After 20 min at 100 °C, the absorbance was read at 532 nm (Jasco v-630, Thermo Fisher Scientific, Tokyo, Japan). The hydroxyl radical scavenging activity was expressed as SC_50_ values (μg GAE/mL).

#### 3.5.2. Antibacterial Activity

##### Bacterial Strain

Methicillin-resistant *S. aureus* strains (INBIOFIV–S1 and S9) were obtained from clinical samples of skin and soft tissue infections from Nestor Kirchner Hospital, San Miguel de Tucumán, Tucumán, Argentina. Methicillin-resistant and -sensitive *S. aureus* ATCC 29213 and ATCC 43300, respectively, were used as controls. All organisms were preserved in brain–heart infusion containing glycerol 30% at −80 °C. The strains were transferred to Mueller Hinton agar (MHA) and incubated at 35 °C for 12 h. Individual colonies were suspended in 5 mL of 0.9% NaCl solution. The cell suspensions were prepared by adjusting turbidity to 0.08 at 560 nm (10^8^ CFU/mL). The cell number in cation-adjusted Mueller–Hinton broth (CAMHB) was estimated using a serial dilution technique, as described in CLSI [83], for each assay.

##### Bioautographic Assay

Plates of silica gel 60 F-254 (0.2 mm, Merck) were seeded with 40 µg of TPC of *S. laevipila* extract. A bioautographic assay was performed using 2 mL of soft medium (BHI with 0.6% agar) containing 10^5^ CFU/mL of methicillin-resistant *S. aureus* (INBIOFIV–S1, INBIOFIV-S9) and ATCC 29213, ATCC 43300. The plates were covered with the inoculated soft medium, incubated at 37 °C for 16–20 h, and then developed with 3-[4, 5-dimethylthiazol-2-yl]-2, 5-diphenyltetrazolium bromide (MTT) solution (2.5 mg/mL) in PBS [84]. The bacterial growth inhibition zones were colored yellow on a bluish background, which showed bacterial growth.

##### Minimal Inhibitory Concentration (MIC)

MIC values of *S. laevipila* extracts were determined using the serial microdilution method. Briefly, 86 µL of Mueller Hinton broth was added to each well of 96-well microplates. Plant dry extract was dissolved in dimethylsulphoxide (DMSO) to obtain a stock solution, and then 4 µL was added to each well containing the medium to obtain a final concentration range of TPC between 200 and 1.8 µg GAE/mL. Subsequently, the culture media were inoculated with 10 µL of a bacterial suspension previously adjusted to an optical density at 560 nm (OD_560nm)_ of 0.08 and then diluted 1:100 to reach a final concentration of 10^5^ CFU/mL. The plates were then incubated at 37 °C for 20 h. Culture medium containing DMSO (60 µL/mL) was used as solvent control. Culture medium without extract and without DMSO was considered as the negative control of inhibition. Different antibiotics were used as positive controls on the INBIOFIV strain collection as described by Leal et al. [85]. The concentrations tested for each antibiotic were those recommended by the CLSI. MIC was defined as the lowest concentration of extract in which the appearance of a button of cells visible to the naked eye was not observed after incubation. The minimum bactericidal concentrations (MBCs) were determined by serial subcultivation of 2 µL of each well without visible growth in Petri dishes with 2 mL of MH medium. The lowest concentration with no visible growth was defined as MBC, indicating 99.5% killing of the original inoculum [86].

### 3.6. Statistical Analysis

All assays were conducted at least three times with three different sample preparations. Each experimental value is expressed as the mean ± standard deviation (SD). The scientific statistic software InfoStat (Student Version, 2011) was used to evaluate the degree of statistical correlation between the different groups [87]. Comparisons between groups were performed by using Pearson’s test.

## 4. Conclusions

In this study, the phytochemical composition of the ethanolic extract of *S. laevipila* was described for the first time, along with its potential use as an antioxidant and antibacterial agent. The moss was collected on *J. mimosifolia*, a tree that grows in the Tucumán Serrano Chaco region. The compounds identified in the extract included both hydrophilic compounds (caffeoyl-D-glucose, kadangustin C, 2’,4’-dihydroxychalcone, 2’,3’-dihydroxychalcone, cirsimaritin, apigenin, chrysoriolin) and non-hydrophilic compounds (hederagenin 20(S), gypsogenin, maslinic acid, mogroside I-A1, recurvoside A, oleanolic acid, bryonoside A, pinellic acid, 9,10-dihydroxy-12-octadecenoic acid, dodecylbenzene sulfonic acid, coriolic acid, *R*-2-hydroxystearic acid, palmitic acid, 6-deoxocastasterone, and holocastasterone), several of which are reported for the first time in the hydroalcoholic extract of mosses. Until now, most chemical studies have focused on vascular plants, with liverworts receiving greater attention, while mosses have been relatively overlooked. However, this study demonstrates that the production of antioxidants and antibacterial extracts from mosses can have a significant impact on the development of cosmetic and pharmaceutical products. As far as we know, some bryophyte-based products are marketed based on the collection of wild populations, which represents a threat to their conservation. Until now, only a few studies on cultivating to generate large amounts of biomass or the production of biomolecules by metabolic engineering have been carried out. Alternative production platforms of bioactive compounds from bryophytes are necessary.

## Figures and Tables

**Table 1:** High-resolution UHPLC-PDA-MS metabolite profiling data of *S. laevipila*.

Peak	Tentative Identification	[M − H]^−^	Retention Time (min.)	Theoretical Mass (m/z)	Measured Mass (m/z)	Accuracy (ppm)	Metabolite Type	MS Ions (ppm)
1	Na formiate (internal standard)	C_4_H_2_O_4_	0.37	112.9829	112.9856	3.1	Standard	
2	Melibiose	C_12_H_21_O_11_	0.77	341.10894	341.10893	−0.0	Sugar	991.9541, 290.08844, 133.01217
3	L-glutamic acid	C_5_H_9_NO_4_	1.12	146.04597	146.05394	0.57	Aminoacid	998.9541
4	Salicylic acid	C_7_H_6_O_3_	6.45	137.02442	137.02441	0.0	Phenolic acid	998.9541
5	Pinellic acid	C_18_H_33_O_5_	7.62	329.23335	329.23252	−2.50	Fatty acids	237.05534
6	2’,4’-Dihydroxychalcone	C_15_H_11_O_3_	8.51	239.07137	239.07021	−4.85	Chalcone	180.9677
7	Caffeoyl -D-Glucose	C_15_H_18_O_9_	9.68	341.1030	341.1041	−6.9	Phenolic acid	191.0513
8	2’,3’-Dihydroxychalcone	C_15_H_11_O_3_	10.01	239.07137	239.07011	−5.27	Chalcone	296.04122, 179.0316
9	Zinniol	C_15_H_22_O_4_	10.22	265.14763	265.14763	11.68	Methoxybencene	150.05522
10	9,10-Dihydroxy-12-octadecenoic acid	C_18_H_33_O_4_	10.73	313.23906	313.23805	0.22	Fatty acid	150.05522
11	Kallolide B	C_13_H_27_O_8_	11.05	311.17114	311.16930	−5.91	Pseudopterane diterpenoid	270.21780
12	Hederagenin	C_30_H_47_O_4_	11.55	471.24798	471.35001	4.29	Triterpenoid	293.21130
13	Asiatic acid	C_30_H_47_O_5_	11.72	487.34290	487.34202	−1.82	Triterpene	291.19983, 267.20309
14	Coriolic acid	C_18_H_31_O_3_	12.05	295.22787	295.22747	−1.36	Fatty acid	269.21455
15	Gypsogenin	C_30_H_45_O_4_	12.18	469.33233	469.33334	−14.4	Triterpenoid	339.20057
16	Maslinic acid	C_30_H_47_O_4_	12.45	471.34798	471.34846	1.00	Triterpenoid	339.20042, 297.24288
17	Piperochromenoic acid	C_22_H_27_O_3_	12.58	339.17993	339.20244	−7.97	Chromene	319.22666, 297.24435
18	Piperochromenoic acid derivative	C_23_H_29_O_3_	13.23	353.21222	353.21538	8.94	Chromene	299.20221, 136.98970
19	5 Alpha-spirostan-3,6 -diol, 6-*O*-Glucoside	C_34_H_57_O_9_	13.31	609.40557	609.40081	7.82	Spirostanol	589.24615
20	Kadangustin C	C_34_H_37_O_11_	13.55	621.24001	621.23826	7.82	Lignans	476.34991, 539.24991
21	Mogroside I-A-1	C_36_H_61_O_9_	14.21	637.43211	637.42216	−15.61	Triterpene	606.24214, 499.33086
22	Recurvoside A	C_35_H_59_O_9_	14.34	623.41646	623.41588	−0.92	Triterpene	473.32190
23	Oleanolic acid	C_30_H_47_O_3_	14.72	455.35307	455.35473	3.64	Triterpene	339.25154
24	Bryonioside A	C_36_H_59_O_9_	15.15	635.41646	635.42096	7.08	Cucurbitane	621.40445, 602.40931, 279.23243
25	(*R*)-2-Hydroxystearic acid	C_16_H_31_O_2_	15.62	299.26188	299.26182	1.24	Fatty acid	169.04162
26	Palmitic acid	C_16_H_31_O_2_	16.02	255.23295	255.23207	−3.48	Fatty acid	169.04162
27	Cirsimaritin	C_17_H_13_O_6_	15.87	313.0717	313.0662	7.8	Flavonoid	271.0798, 627.14120 (2M-H)-270.0795
28	6-Deoxocastasterone	C_28_H_47_O_4_	16.13	447.34798	447.34829	0.68	Brassinosteroid	307.13505
29	Apigenin	C_15_H_10_O_5_	16.45	269.04568	269.04554	0.48	Flavonoid	179.0318
30	Dictamnin A	C_36_H_59_O_8_	17.17	619.42165	619.42155	1.62	Alkaloid	577.43479
31	Chrysoeriol	C_16_H_12_O_6_	17.26	299.0502	299.0520	4.2	Flavonoid	271.0550
32	Homocastasterone	C_29_H_49_O_5_	17.52	477.35855	477.35693	−3.39	Brassinosteroid	455.01804
33	Panaxynol linoleate	C_35_H_53_O_2_	18.75	505.39753	505.40510	−14.98	Triterpene	414.99256

**Table 2:** Pearson correlation coefficients of the content of total phenolics, flavonoids, and antioxidant capacity determined by different methods.

	TPC	TF	ABTS^•+^	H_2_O_2_	HO•	AAPH
TPC	1.00	0.20	0.30	0.11	0.23	0.07
TF	0.95	1.00	0.09	0.09	0.03	0.13
ABTS^•+^	0.89	0.99	1.00	0.18	0.06	0.23
H_2_O_2_	0.98	0.99	0.96	1.00	0.12	0.04
HO•	0.93	1.00	1.00	0.98	1.00	0.17
AAPH	0.99	0.98	0.94	1.00	0.97	1.00

TPC: total phenolic compounds; TF: total flavonoids; ABTS^•+^: ABTS cation radical; HO• (hydroxyl radical), H_2_O_2_, AAPH: 2,2’-Azobis(2-amidinopropane) dihydrochloride.

**Table 3:** Antimicrobial activity of *S. laevipila* extract against clinical isolates and ATCC strain and resistance profile of *S. aureus*.

*S. aureus* Strain	MIC µg/mL	Phenotype of *S. aureus* (MIC μg/mL)
INBIOFIV-S1	3.7	LEV^S^ (CIM ≤ 1), MET^R^, (CIM ≥ 4), GEN^R^ (CIM ≥ 16)
(CIM ≥ 16)INBIOFIV-S9	3.7	LEV^R^, (CIM ≥ 4), MET^R^ (CIM ≥ 4), GEN^R^, (CIM ≥ 16)
ATCC 43300	7.5	MET^R^ (CIM ≥ 4)
ATCC 29213	7.5	MET^S^ (CIM = 0.25)

MIC: minimal inhibitory concentration. Levofloxaxina (LEV), methicillin (MET), gentamycin (GEN). R: resistant; S: sensitive.

## Data Availability

The original contributions presented in the study are included in the article/Supplementary Material, further inquiries can be directed to the corresponding author.

## Supplementary Materials

The following supporting information can be downloaded at: https://www.mdpi.com/article/10.3390/plants14020253/s1, File S1: Q TOF-MS and MSn spectra of some representative compounds and standard curves.
