# Supplementary material for: Syntrichia laevipila Brid., a Bryophyta from Northwest Argentina as a Source of Antioxidants and Antimicrobials
Source: Plants (Basel). 2025 Jan 17;14(2):253. doi: 10.3390/plants14020253 (PMC11768277; doi:10.3390/plants14020253)

Supplementary material

Q TOF-MS and MSn spectra of some representative compounds and standard curves.

Peak 5: Pinellic acid

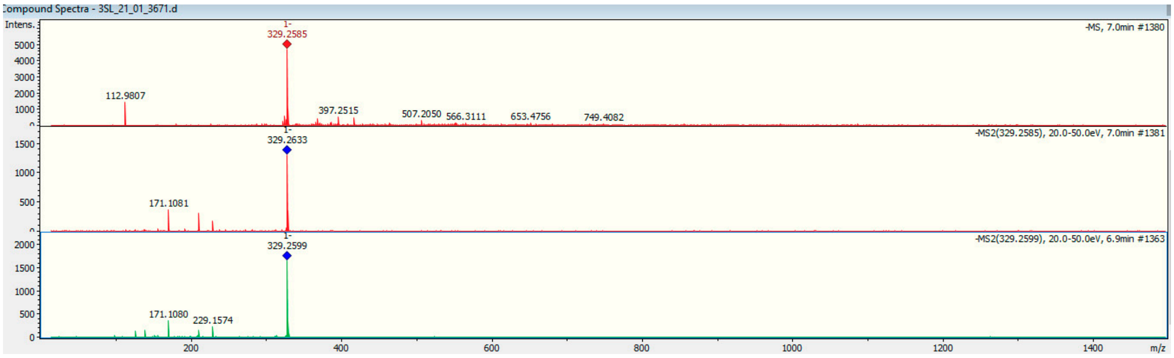

Peak 6: 2',4'- Dihydroxychalcone

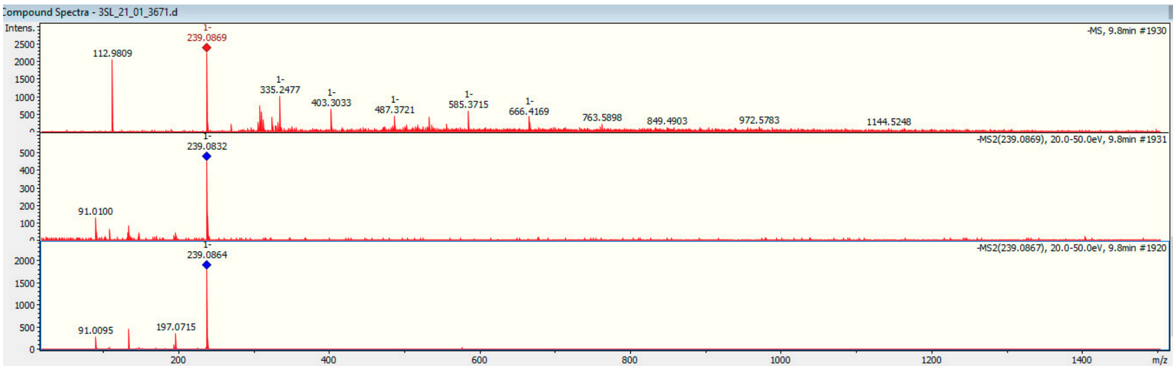

Peak 12: Hederagenin

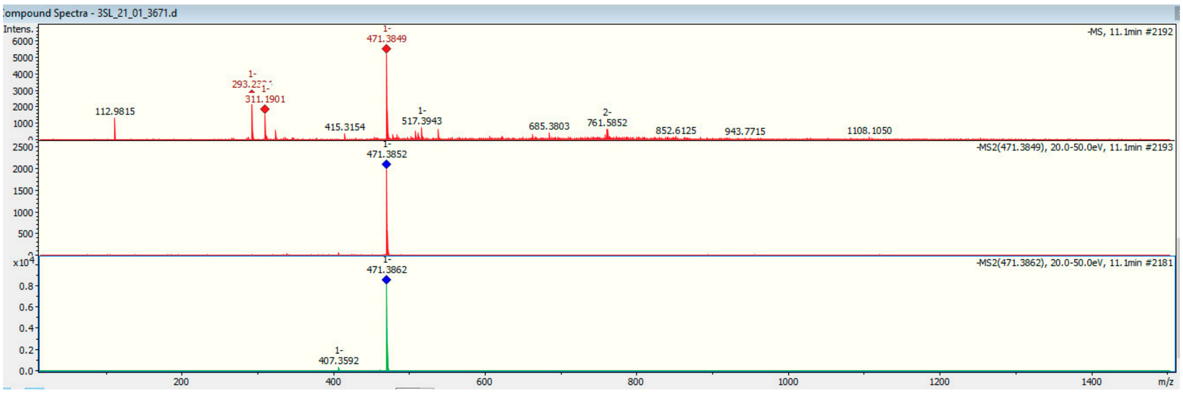

Peak 16: Maslinic acid

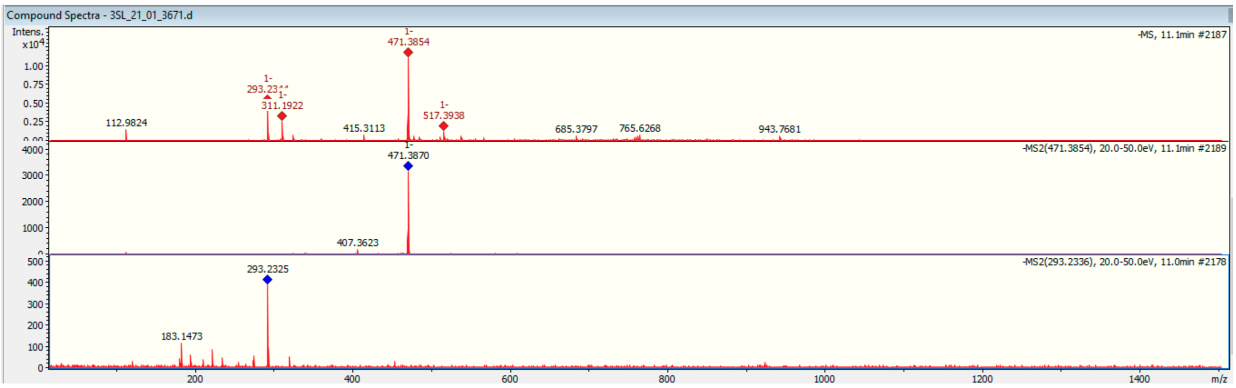

Peak 17: Piperochromenoic acid

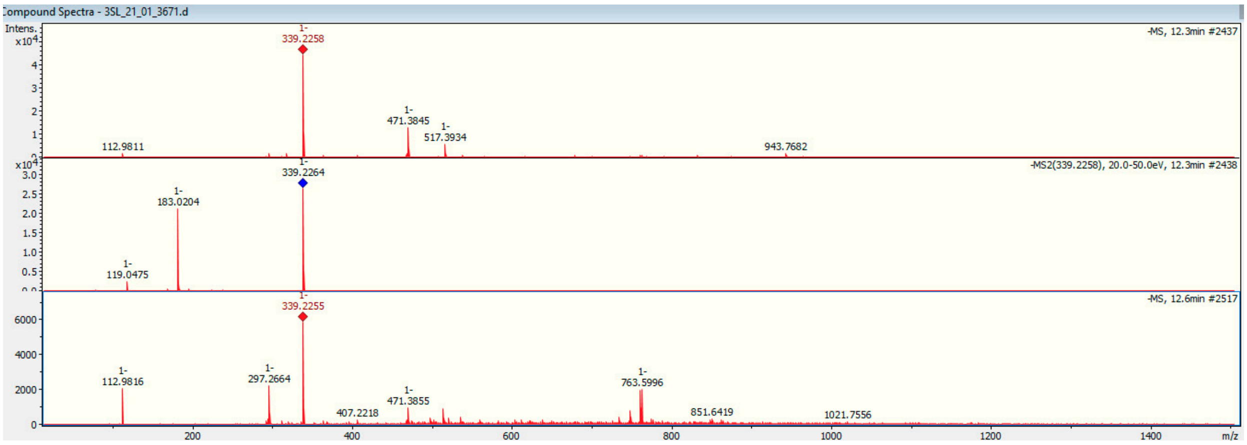

Peak 21: Mogroside I-A-1

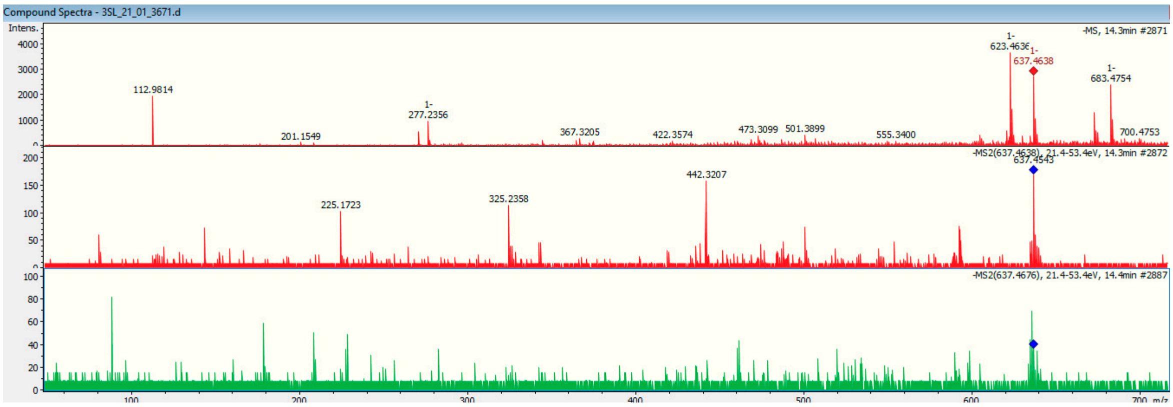

Peak 22: Recurvoside A

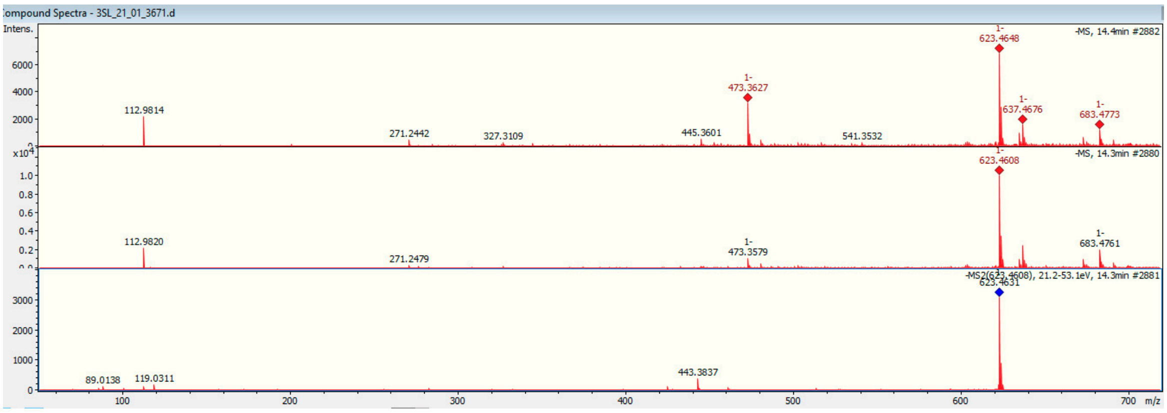

Peak 23: Oleanolic acid

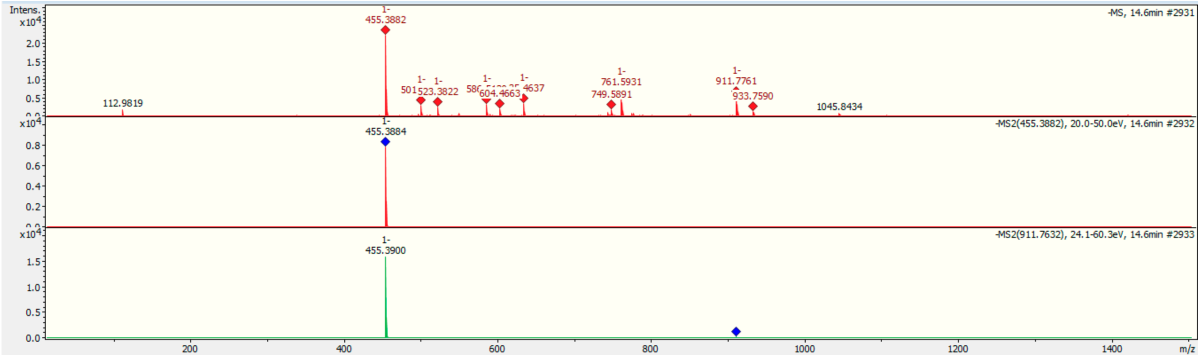

Peak 27: Cirsimaritin

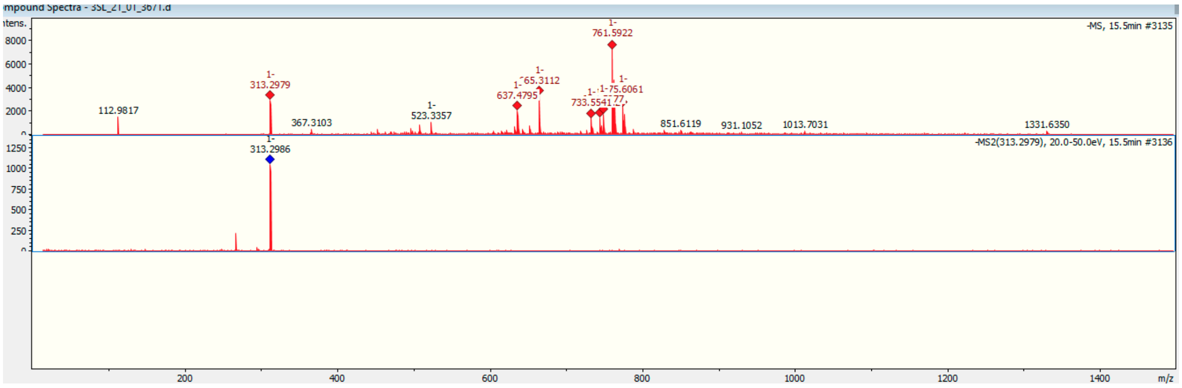

Total phenolic compounds content by reaction with Folin–Ciocalteu reagent

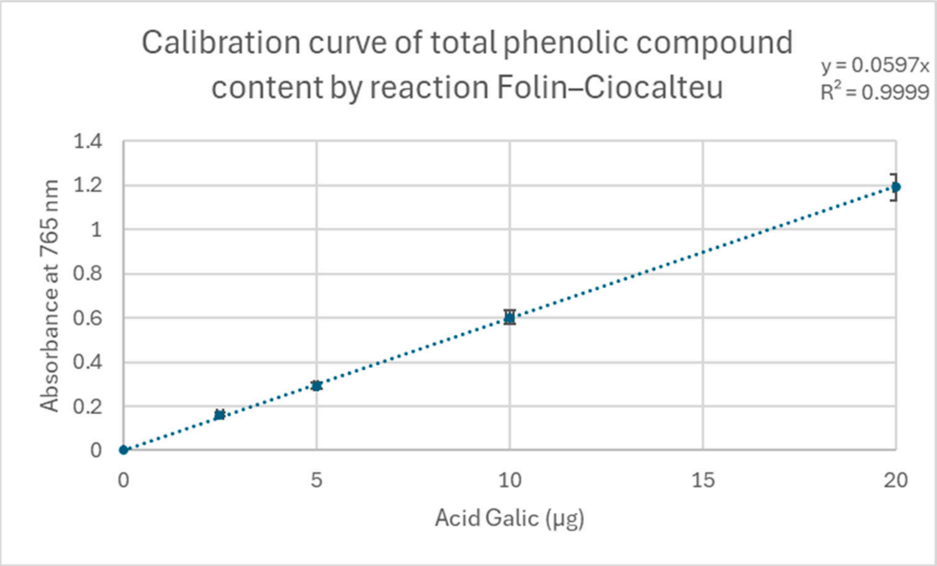

Total flavonoids content by reaction with aluminum chloride

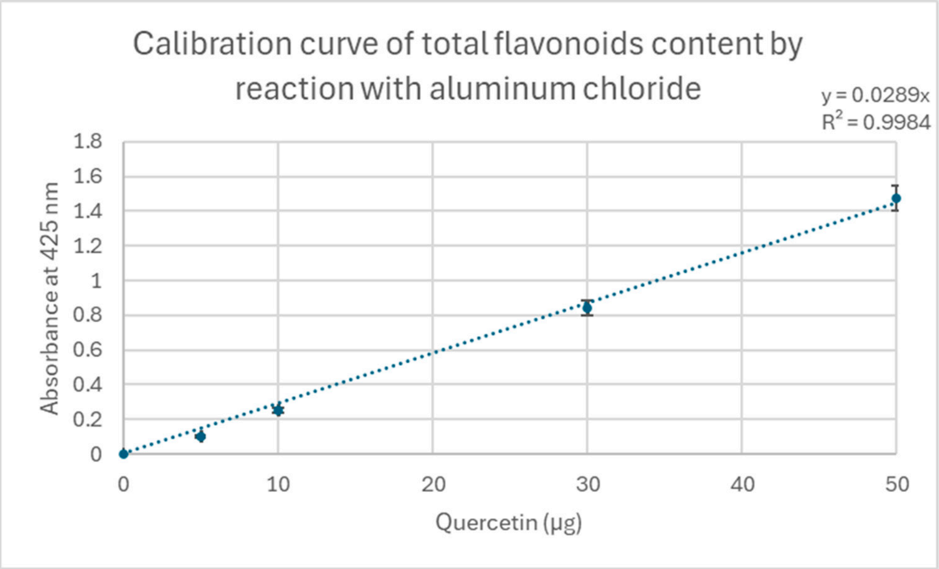

Supplement: Supplementary file 1 [file plants-14-00253-s001.zip › plants-3374615-supplementary.pdf]
